# Association between alcohol-Induced mental and behavioral disorders and comorbidities: Evidence from the Korea National Hospital Discharge In-depth Injury survey data

**DOI:** 10.1371/journal.pone.0356598

**Published:** 2026-09-16

**Authors:** Jieun Hwang, Seri Kang, Hyunkyung Lee, Kyunghee Lee

**Affiliations:** 1 College of Health Science, Dankook University, Dongnam-gu, Cheonan city, Chungcheongnam-do, South Korea; 2 Department of Healthcare Management, Eulji University of Korea, Sujeong-gu, Seongnam city, Kyeonggi-do, South Korea; 3 Department of Health management, Jeonju University, Wansan-gu, Jeonju city, Jeollabuk-do, South Korea; Jawaharlal Institute of Postgraduate Medical Education and Research, INDIA

## Abstract

**Background:**

Alcohol consumption is a significant social medium in South Korea, yet alcohol use disorders (AUD) impose a substantial public health burden. This study aimed to analyze sex-specific clinico-demographic characteristics, comorbidity patterns, and factors affecting the length of stay (LOS) among patients hospitalized for alcohol-induced mental and behavioral disorders using a nationally representative database.

**Methods:**

This retrospective cross-sectional study utilized data from the Korea National Hospital Discharge In-Depth Injury Survey (KNHDIS) from 2014 to 2023. After applying sampling weights to account for the complex survey design, a total of 201,572 patients were included in the analysis. Differences in general characteristics were tested using the Rao-Scott chi-square test, and factors influencing log-transformed LOS were identified through multiple linear regression analysis.

**Results:**

Demographic Profile: Male patients were most prevalent in their 50s (36.6%), whereas female patients showed a younger age distribution, peaking in their 40s (31.1%). Comorbidity Patterns: Men had a higher prevalence of cardiovascular disease (30.0%) and diabetes (20.2%). Conversely, women exhibited significantly higher rates of comorbid mental disorders (31.5%) and injuries (19.8%). Predictors of LOS: Comorbid mental disorders were the strongest predictors of prolonged LOS in both sexes. However, injuries and neurological diseases significantly extended LOS only in men, showing no significant impact in women.

**Conclusions:**

Comorbidity patterns and medical resource utilization patterns among patients with alcohol use disorder differ markedly by sex. Therefore, alcohol addiction management policies and clinical interventions require sex-tailored approaches, particularly emphasizing the urgent establishment of multidisciplinary integrated treatment systems for cases with comorbid mental health disorders.

## Introduction

### Background and significance of the study

In Korean society, alcohol consumption is perceived as a primary facilitator of social bonding—extending beyond a mere act of personal indulgence to serve as a medium for professional gatherings, social interactions, celebrations, and emotional support. This cultural milieu tends to normalize drinking as an indispensable daily activity, which has led to persistent concerns regarding the promotion of excessive or harmful drinking [1,2]. Notably, recent studies indicate a significant increase in drinking frequency among younger populations and a rising trend in high-risk drinking specifically within women [2–4]. These phenomena impose substantial socioeconomic burdens, including escalating healthcare costs, diminished productivity, and links to crime or accidents. Consequently, alcohol-related public policies—such as taxation, alcohol availability restrictions, and enhanced penalties for driving under the influence (DUI)—have emerged as critical public health priorities [3,5,6].

While alcohol consumption is a ubiquitous lifestyle habit globally, the resulting health burden is becoming increasingly severe. According to the World Health Organization (WHO), harmful alcohol use was responsible for approximately 3 million deaths in 2016—representing 5.3% of all global fatalities—with a significant proportion of these deaths occurring among young adults aged 20–39 years (World Health Organization, 2018).

According to previous domestic studies, the drinking rate among Korean adults (aged 19 years and older) is remarkably high at approximately 74.8%, with the prevalence of high-risk drinking being particularly pronounced among men and middle-aged individuals [1,7]. Specifically, the prevalence and incidence of alcoholic liver disease (ALD) vary according to drinking patterns, with high-risk drinkers exhibiting a considerably higher likelihood of developing ALD. Furthermore, it has been reported that women among high-risk drinkers show a higher risk ratio for ALD compared to their male counterparts [2].

Despite these high prevalence rates and associated health risks, the proportion of individuals reporting alcohol consumption at least once a month has consistently exceeded 50% over the past decade [8], suggesting that alcohol consumption is extensively internalized throughout society. Alcohol use serves as a major risk factor not only for various physical diseases—including liver disease, cardiovascular disease, and cancer—but also for serious mental health issues, such as alcohol-related mental and behavioral disorders [3].

According to domestic studies, alcohol dependence not only induces physical complications such as liver disease, hypertension, and diabetes, but also exhibits a remarkably high comorbidity rate with various mental disorders, including depression and anxiety disorders [9,10]. International research has consistently demonstrated that alcohol use disorder (AUD) is closely linked to mental health, with a high proportion of AUD patients exhibiting comorbid conditions such as depression, anxiety disorders, and other psychiatric illnesses [11]. Furthermore, multinational comparative studies have reported that the prevalence of AUD (lifetime or past-year) varies considerably among nations depending on cultural and socioeconomic factors, and that the presence of comorbid mental disorders substantially affects treatment costs, recovery prognosis, and the resulting social burden [12].

Globally, research on the health effects of alcohol use has been continuously conducted [13,14], and in Korea, the Korea Disease Control and Prevention Agency (KDCA) annually releases indicators such as the 13 disease categories associated with alcohol-related mortality and the mortality rate attributable to drinking [15]. However, empirical data on hospitalizations resulting from mental and behavioral disorders or the current status of comorbidities remain insufficient. This lack of data raises concerns that both the justification of healthcare service provision and the efficiency of resource allocation may be compromised.

In addition, domestic research has primarily focused on analyzing the physical health impacts of alcohol consumption, with relatively limited investigation into the comorbid associations or influencing factors related to specific types of mental disorders. Identifying the specific patterns of comorbidities would provide medical institutions with the necessary evidence to identify target populations and develop interdisciplinary treatment strategies. For instance, for patients with alcohol use disorder (AUD) comorbid with depression or anxiety disorders, strengthening the linkage with mental health services should be prioritized; similarly, in cases involving liver disease or trauma, the need for early diagnosis and intervention could be more specifically defined.

Ultimately, policy interventions that focus solely on alcohol consumption without an adequate understanding of comorbid conditions risk exacerbating a confluence of issues, including escalating medical costs, diminished quality of life, premature mortality, and the loss of labor productivity. Therefore, this study aims to systematically identify the current status of comorbidities among patients with mental and behavioral disorders due to alcohol use and analyze their related factors. By doing so, the study seeks to provide foundational data to inform clinical interventions and policy development.

### Research objectives

This study utilized data from the National Hospital Discharge In-depth Injury Survey collected by the Korea Disease Control and Prevention Agency (KDCA) to analyze the sociodemographic and clinical characteristics, as well as the prevalence patterns of comorbidities, among patients diagnosed with alcohol-related mental and behavioral disorders (ICD-10 codes F10.0–F10.9). The findings are intended to provide foundational data for the development of mental health promotion and addiction management policies, while also establishing an evidentiary basis to support interdisciplinary treatment approaches within medical institutions for individuals with these disorders.

The specific objectives of this study are as follows:

(1) To examine the sociodemographic and hospitalization characteristics of patients with alcohol-related mental and behavioral disorders using data from the National Hospital Discharge In-depth Injury Survey.
(2) To determine the specific types and prevalence of comorbidities among patients diagnosed with alcohol-related mental and behavioral disorders.
(3) To identify the associations between alcohol-related mental and behavioral disorders (ICD-10 codes F10.0–F10.9) and their comorbid conditions.

## Materials and methods

### Data source

This retrospective cross-sectional study utilized data from the Korean National Hospital Discharge In-Depth Injury Survey (KNHDIS) conducted by the Korea Disease Control and Prevention Agency (KDCA) [16]. Launched in 2005, this survey is a nationally representative probability sample survey that provides the fundamental data necessary to support the development of national health policies and injury prevention strategies. KNHDIS employs a two-stage stratified cluster sampling method to select approximately 9% of discharge cases annually from general hospitals with ≥100 beds, stratified by bed size (100–299, 300–499, 500–999, ≥ 1,000) and geographic location.

This study was approved by the Institutional Review Board of Eulji University (IRB No. EUIRB2025−338). The IRB waived the requirement for informed consent as this study is a retrospective analysis of de-identified secondary data. For the purposes of this research, the authors accessed the data on January 27, 2026. Throughout the study, the authors had no access to any information that could identify individual participants, as the KNHDIS dataset is provided by the KDCA in a fully de-identified and anonymized format to ensure participant confidentiality.

### Study population

**Fig 1:**
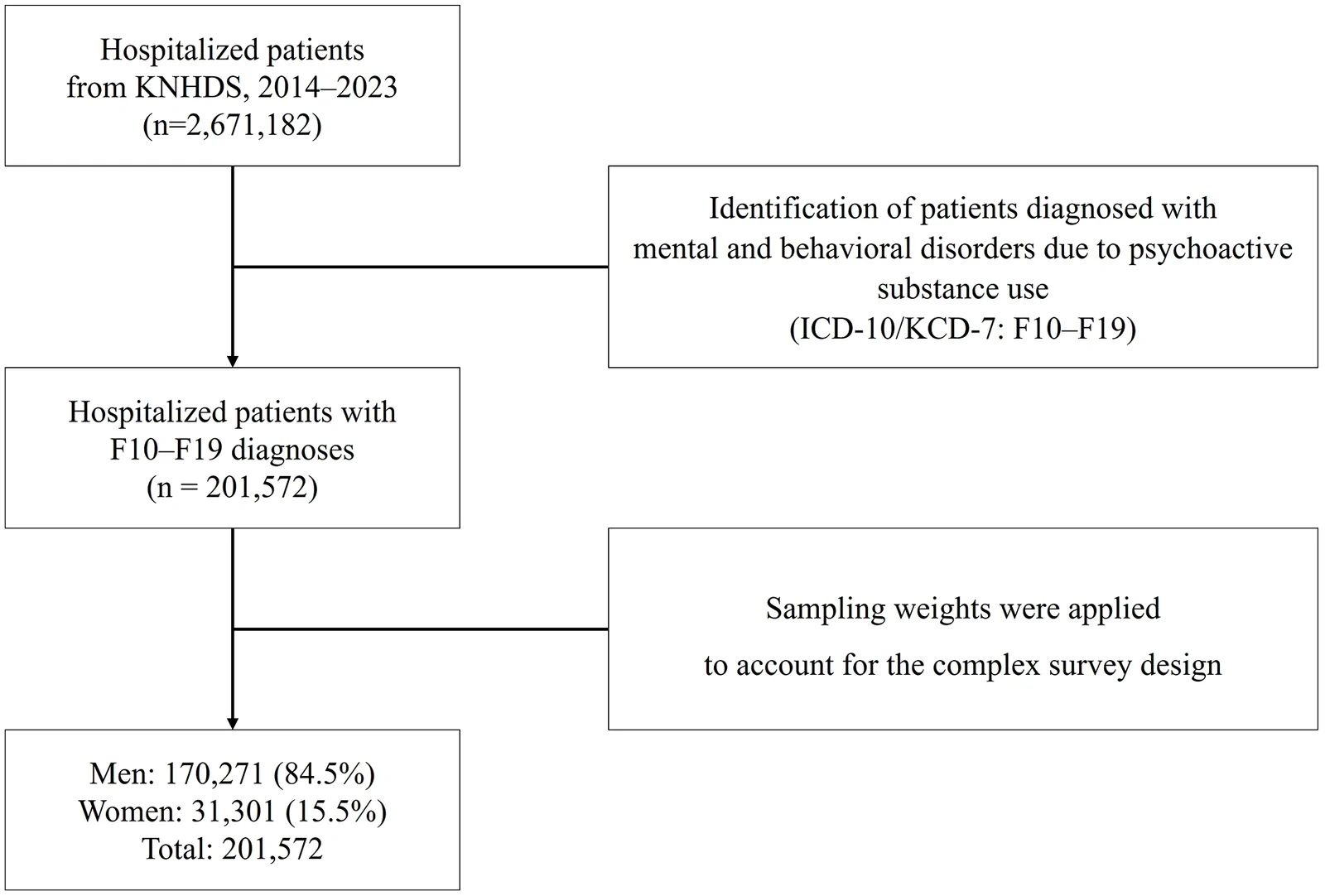
Flowchart for study data selection.

Fig 1 illustrates the flowchart of patient selection from the KNHDIS database (2014–2023). Patients discharged with principal or secondary diagnoses of “mental and behavioral disorders due to use of alcohol” (F10.0–F10.9; ICD-10/KCD) were identified. After applying sampling weights to account for the complex survey design, a total of 201,572 patients were included in the final analysis (170,271 men [84.5%]; 31,301 women [15.5%]).

### Study variables

#### Dependent variable.

The length of stay (LOS), defined as the total duration of hospitalization, was used as an indicator of healthcare resource consumption. LOS was calculated as the number of days from admission to discharge, with same-day discharges assigned a LOS of one day, consistent with standard conventions in Korean national hospital discharge data analyses and to prevent a LOS value of zero which would be undefined under logarithmic transformation. To correct the non-normal distribution of the LOS data and to improve the fit of the regression model, the natural logarithm (log) of LOS was used as the final dependent variable in the analysis.

#### Independent variables.

In this study, patients diagnosed with mental and behavioral disorders due to alcohol use (F10) according to International Classification of Diseases, Tenth Revision (ICD‑10) and the Korean Standard Classification of Diseases (KCD) were described in the context of Alcohol Use Disorder (AUD) as defined in the Diagnostic and Statistical Manual of Mental Disorders, 5th Edition (DSM‑5). Comorbidity was defined as the presence of specific conditions co-occurring with a diagnosis of AUD (F10). The primary comorbidities analyzed included cardiovascular diseases (I00–I99), diabetes mellitus (E10–E14), liver diseases (K70–K77), neurological disorders (G00–G99), injuries (S00–T98), and mental and behavioral disorders excluding F10 (F00–F99). Each comorbidity was treated as a binary variable (present/absent) for analysis.

#### Control variables.

The demographic characteristics of the participants included sex and age groups (<30, 30–39, 40–49, 50–59, 60–69, and ≥70 years). Healthcare utilization and policy-related factors included the payment method (National Health Insurance, Medical Aid, and Other), hospital bed size (100–299, 300–499, 500–999, and ≥1,000 beds), and discharge outcome (Improved, Not improved, In-hospital mortality, and Other). Additionally, the discharge year was included as a control variable to adjust for annual trends.

### Analysis methods

This study employed complex sample analysis techniques that incorporated the weight, strata, and cluster variables in the analysis to account for the complex survey design of the Korean National Hospital Discharge In-depth Injury Survey.

First, weighted frequencies and weighted percentages were calculated by sex to describe the participants’ general characteristics, yearly distribution, and distribution of comorbidities. Differences in the distribution of each variable by sex were tested for statistical significance using the Rao-Scott chi-square test.

Second, the weighted mean length of stay (LOS) and standard error (SE) were calculated and compared to examine differences in healthcare resource utilization by sex and other major characteristics.

Third, given the well-documented sex differences in the prevalence, comorbidity profile, and clinical presentation of alcohol use disorder, gender-stratified multiple linear regression models were constructed separately for male and female patients to identify sex-specific predictors of length of stay (LOS). To mitigate the skewness of the LOS data and ensure the normality of the residuals in the regression model, the dependent variable (LOS) was log-transformed. Age group, payment method, treatment outcome, bed size, discharge year, and diagnosis subcategory (F10.0–F10.9) were adjusted for as control variables in the analysis. To facilitate clinical interpretation, the log-transformed regression coefficients were back-transformed using the formula (e^B − 1) × 100% to express the effect of each comorbidity as a percentage change in LOS.

Multicollinearity among the six comorbidity indicators was assessed using the Variance Inflation Factor (VIF) prior to model fitting; all VIF values were below 1.3 (range, 1.03–1.23) in both the male and female models, indicating no substantial multicollinearity among the comorbidity variables of primary interest

All analyses were performed using SAS 9.4 (SAS Institute Inc., Cary, NC, USA), with statistical significance level set at p < .05.

## Results

### Trends and general characteristics of alcohol use disorder patients

**Fig 2:**
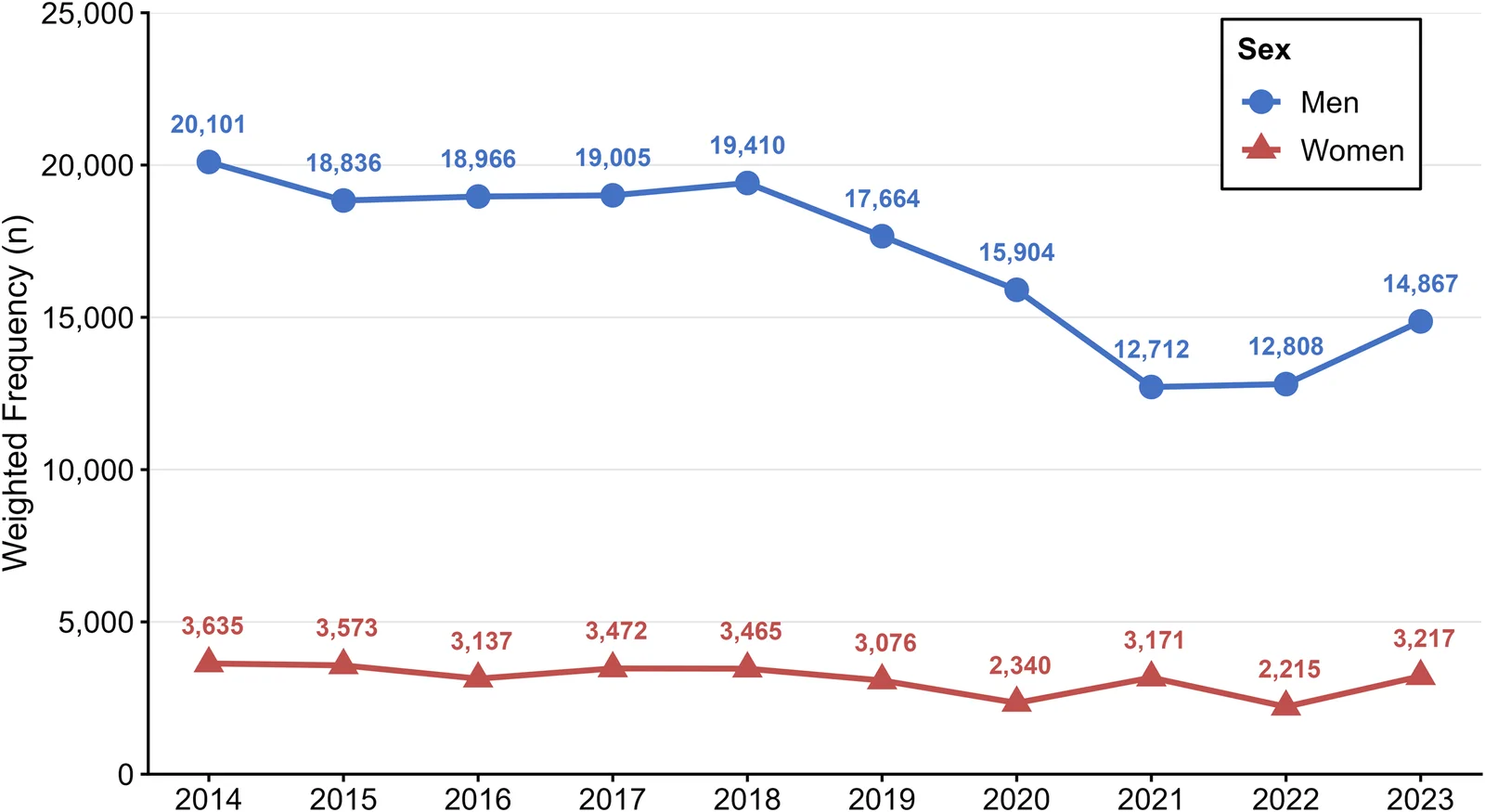
Changes in weighted frequency of alcohol-use disorders by sex (2014–2023).

Fig 2 presents the changes in the weighted frequency of patients discharged with alcohol-related mental and behavioral disorders by sex over the past decade (2014–2023). For men, the highest number of discharges was recorded in 2014 (20,101 cases), followed by moderate fluctuations until 2018. However, a pronounced declining trend began in 2019, reaching a ten-year low in 2021 (12,712 cases). Subsequently, the frequency shifted back to an upward trend, reaching 14,867 cases in 2023. Regarding women, while the overall frequency remained consistently lower than that of men, it showed minor fluctuations until the mid-2010s. After reaching its lowest point in 2022 (2,215 cases), the frequency rebounded in 2023 (3,217 cases). The declining trend observed from 2020 to 2022 suggests the impact of the COVID-19 pandemic [17].

Table 1 presents the sociodemographic characteristics of the 201,572 patients discharged with alcohol-related mental and behavioral disorders over the past decade (2014–2023). Specifically, men exhibited the highest proportions in their 50s (36.6%) and 60s (22.1%), whereas those of women were predominantly in their 40s (31.1%) and 50s (21.3%), indicating a relatively younger age distribution among female patients (p < .0001). No statistically significant difference was observed in the type of payment between sexes (p = .0982). At discharge, the improvement rate was very high for both groups (91.6% for men and 92.7% for women; p = 0.2359). In contrast, significant differences were observed in the utilization of hospitals by bed capacity. While the highest proportion of male patients (40.0%) utilized hospitals with 100–299 beds, female patients showed a higher utilization rate (40.1%) for hospitals with 500–999 beds (p = .0089).

**Table 1:** General characteristics of patients with mental and behavioral disorders due to use of alcohol by sex 2014–2023.

Category	Variable	Men (n = 170,271)		Women (n = 31,301)		p-value
		n	%	n	%	
Age Group	<30	2,693	1.6	2,471	7.9	<.0001
	30s	10,108	5.9	6,475	20.7	
	40s	37,383	22.0	9,735	31.1	
	50s	62,335	36.6	6,673	21.3	
	60s	37,670	22.1	3,574	11.4	
	≥70	20,082	11.8	2,374	7.6	
Type of Payment	National Health Insurance	122,387	71.9	23,539	75.2	0.0982
	Medical Aid	45,959	27	7,373	23.6	
	Other	1,925	1.1	389	1.2	
Discharge Status	Improved	155,986	91.6	29,026	92.7	0.2359
	Not Improved	7,785	4.6	1,434	4.6	
	Other	2,163	1.3	388	1.2	
	In-hospital mortality	4,337	2.5	453	1.4	
Hospital Size	100-299 beds	68,183	40	10,880	34.8	0.0089
	300-499 beds	28,786	16.9	5,363	17.1	
	500-999 beds	59,215	34.8	12,539	40.1	
	1000 beds	14,087	8.3	2,519	8.0	

Note: n = weighted frequency; % = weighted percentage; p-values were calculated using the Rao-Scott chi-square test.

### Distribution of specific diagnosis of alcohol-related mental and behavioral disorders by sex

**Fig 3:**
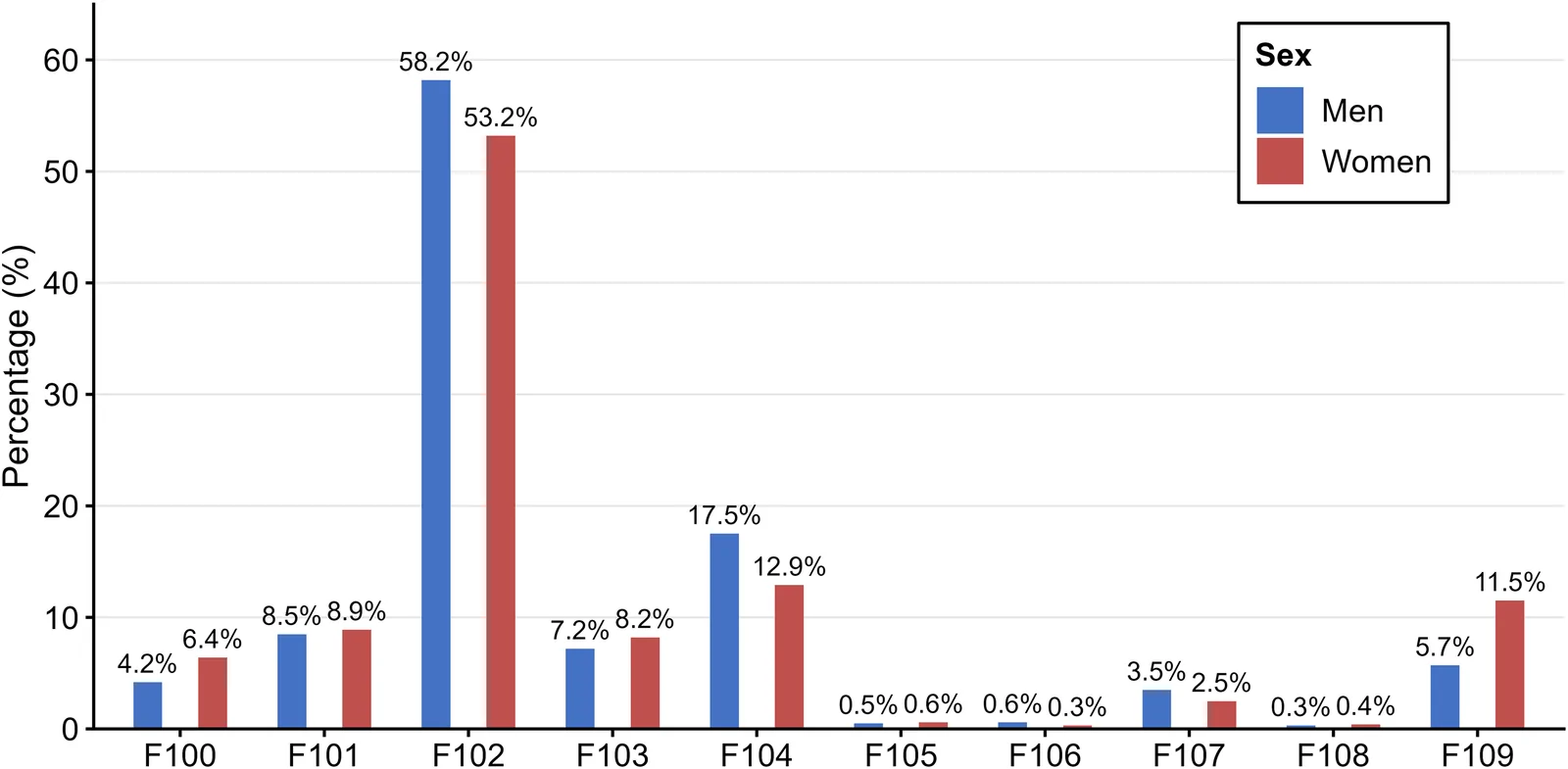
Distribution of percentages for specific diagnoses of alcohol-induced mental and behavioral disorders by sex.

The analysis of the sex-specific diagnosis distribution for patients discharged with alcohol-related mental and behavioral disorders (including multiple diagnoses) is shown in Fig 3. The analysis revealed that dependence syndrome (F10.2: Mental and behavioral disorders due to use of alcohol: dependence syndrome) was the most prevalent diagnosis across both sexes. Specifically, 58.2% of men and 53.2% of women had dependence syndrome, indicating that more than one in two patients discharged with alcohol-related disorders were diagnosed with a state of dependence.

An analysis of sex differences revealed that the withdrawal state with delirium (F10.4: Mental and behavioral disorders due to use of alcohol: withdrawal state with delirium), indicative of severe withdrawal symptoms, was more prevalent among men (17.5%) than women (12.9%). In contrast, unspecified mental and behavioral disorder (F10.9: Mental and behavioral disorders due to use of alcohol: unspecified mental and behavioral disorder) showed approximately twice the prevalence in women (11.5%) compared to men (5.7%). This finding suggests that female patients may exhibit atypical patterns that are more challenging to classify into specific diagnostic categories, or that unspecified codes may be more extensively utilized due to overlapping symptoms.

### Comorbidity patterns by sex

Table 2 presents the prevalence of comorbidities among patients with alcohol-related mental and behavioral disorders by sex. Cardiovascular diseases (30.0% in men, 21.8% in women) and diabetes (20.2% in men, 11.0% in women) showed statistically significantly higher prevalence in men (p < .0001). In contrast, trauma (14.3% in men, 19.8% in women; p = 0.002) and mental disorders (21.7% in men, 31.5% in women; p < .001) exhibited significantly higher prevalence in women. Liver diseases recorded the highest comorbidity rates in both groups (43.3% in men, 42.4% in women), but the sex difference was not statistically significant (p = 0.6234). Similarly, no significant difference was observed for neurological disorders (13.7% in men, 12.3% in women; p = 0.2588).

**Table 2:** Prevalence of comorbidities among patients with alcohol-induced mental and behavioral disorders by sex.

Category		Presence of comorbidity	Men (n = 170,271)		Women (n = 31,301)		p-value
			n	%	n	%	
Cardiovascular Disease	(I00-I99)	absent	119,159	70.0	24,492	78.2	<.0001
		present	51,112	30.0	6,809	21.8	
Diabetes Mellitus	(E10-E14)	absent	135,920	79.8	27,863	89.0	<.0001
		present	34,351	20.2	3,438	11.0	
Liver Disease	(K70-K77)	absent	96,507	56.7	18,043	57.6	0.6234
		present	73,764	43.3	13,258	42.4	
Neurological Disease	(G00-G99)	absent	146,866	86.3	27,440	87.7	0.2588
		present	23,405	13.7	3,861	12.3	
Injury	(S00-T98)	absent	145,942	85.7	25,104	80.2	0.0002
		present	24,329	14.3	6,197	19.8	
Mental Disorder	(F00-F99, Excluding F10)	absent	133,303	78.3	21,457	68.6	<.0001
		present	36,968	21.7	9,844	31.5	

Note: n = weighted frequency; % = weighted percentage; p-values were calculated using the Rao-Scott chi-square test.

### Comparison of mean length of stay by sex and patient characteristics

Table 3 presents the comparison of the mean length of stay (LOS) by major patient characteristics and sex among patients with alcohol-related mental and behavioral disorders. The analysis revealed no statistically significant differences in mean LOS between sexes across age groups, treatment outcomes, or hospital bed size (p > .05). In contrast, the LOS varied significantly by type of payment, with male patients exhibiting longer hospital stays than female patients. Particularly among Medical Aid patients, men (13.2 ± 0.4) had a longer mean LOS than women (11.8 ± 0.7) (p = 0.0498).

**Table 3:** Comparison of mean length of stay by sex and general and clinical characteristics.

Category	Variable	Presence of comorbidity	Men		Women		p-value
			Mean	SE	Mean	SE	
Age group	<30	–	7.8	0.9	11.1	1.2	0.1167
	30s	–	11.3	0.6	11.4	0.7	
	40s	–	11.6	0.4	12.2	0.7	
	50s	–	12.4	0.3	11.9	0.8	
	60s	–	12.0	0.4	10.4	0.9	
	≥70	–	11.7	0.4	11.6	1.4	
Type of payment	National Health Insurance	–	11.3	0.2	11.6	0.4	0.0498
	Medical Aid	–	13.2	0.4	11.8	0.7	
	Other	–	16.6	2.2	11.3	2.6	
Discharge status	Improved	–	12.2	0.2	11.9	0.4	0.2694
	Not Improved	–	7.4	0.8	7.5	1.1	
	Other	–	5.4	0.8	9.6	2.5	
	In-hospital mortality	–	12.5	1.2	8.9	3.3	
Hospital size	100-299 beds	–	11.8	0.4	10.4	0.7	0.0834
	300-499 beds	–	12.7	0.4	14.1	1.2	
	500-999 beds	–	11.8	0.3	11.5	0.4	
	1000 beds	–	10.9	0.4	12.4	0.9	
Comorbidities							
Cardiovascular disease	(I00-I99)	absent	11.6	0.2	11.2	0.4	0.2102
		present	12.5	0.3	13.3	0.8	
Diabetes mellitus	(E10-E14)	absent	11.5	0.2	11.4	0.4	0.9483
		present	13.3	0.4	13.1	1.2	
Liver disease	(K70-K77)	absent	11.7	0.3	11.0	0.5	0.2442
		present	12.2	0.2	12.4	0.5	
Neurological disease	(G00-G99)	absent	11.4	0.2	11.5	0.4	0.0367
		present	15.1	0.5	12.7	1.0	
Injury	(S00-T98)	absent	11.3	0.2	11.6	0.4	0.0008
		present	15.3	0.5	11.8	0.9	
Mental disorder	(F00-F99, Excluding F10)	absent	11.0	0.2	10.2	0.4	0.5751
		present	15.2	0.4	14.9	0.8	

Note: Values are mean length of stay (days) with standard error (SE); -, not applicable

Significant sex differences in length of stay (LOS) were observed depending on the presence of comorbidities, particularly for neurological disorders and trauma. Among patients with comorbid neurological disorders, men had a significantly longer mean LOS of 15.1 ± 0.5 days compared to 12.7 ± 1.0 days for women (p = 0.0367). Similarly, among those with trauma, men exhibited a mean LOS of 15.3 ± 0.5 days, which was substantially longer than the 11.8 ± 0.9 days observed in women (p = 0.0008). No significant sex differences in LOS were found for patients with cardiovascular diseases, diabetes, liver diseases, or mental disorders (p > .05).

### Analysis of the factors influencing length of stay due to comorbidities

Table 4 presents the results of the analysis regarding the effects of comorbidities on the length of stay (LOS) among patients with alcohol use disorder.

**Table 4:** Factors associated with length of stay: linear regression analysis by sex.

Category	Men				Women			
	B	95% CI	t Value	p-value	B	95% CI	t Value	p-value
Cardiovascular Disease	0.070	(0.010-0.130)	2.28	0.0229	0.219	(0.049-0.389)	2.53	0.0119
Diabetes Mellitus	0.144	(0.080-0.209)	4.38	<.0001	0.196	(0.006-0.387)	2.02	0.0438
Liver Disease	0.111	(0.052-0.169)	3.72	0.0002	0.236	(0.102-0.371)	3.45	0.0006
Neurological Disease	0.267	(0.186-0.348)	6.44	<.0001	0.107	(-0.098-0.311)	1.03	0.3059
Injury	0.348	(0.271-0.424)	8.93	<.0001	0.081	(-0.083-0.246)	0.97	0.333
Mental Disorder	0.356	(0.279-0.433)	9.08	<.0001	0.350	(0.195-0.506)	4.43	<.0001

Note: B: unstandardized coefficient; CI: confidence interval; Adjusted for age group, payment method, treatment results, hospital size, discharge year and diagnosis subcategory (F10.0–F10.9); Reference group for each comorbidity is ‘No.’

The analysis revealed that for male patients, all comorbidities were significantly associated with an increase in LOS (p < .05). Specifically, mental disorders (B = 0.356, p < .0001; a 42.8% increase in LOS) and injury (B = 0.348, p < .0001; a 41.6% increase) were found to have the most substantial impact on extending LOS. These were followed by neurological disorders (B = 0.267, p < .0001; a 30.6% increase), diabetes mellitus (B = 0.144, p < .0001; a 15.5% increase), liver diseases (B = 0.111, p = .0002; an 11.7% increase), and cardiovascular diseases (B = 0.070, p = .0229; a 7.3% increase), all of which significantly prolonged the duration of hospitalization.

In female patients, certain disease groups showed significant impacts on increased length of stay (LOS), exhibiting different patterns compared to male patients. Similar to men, comorbid mental disorders (B = 0.350, p < .0001; a 41.9% increase in LOS) were identified as a factor prolonging LOS in women. Liver diseases (B = 0.236, p = .0006; a 26.6% increase), cardiovascular diseases (B = 0.219, p = .0119; a 24.5% increase), and diabetes mellitus (B = 0.196, p = .0438; a 21.7% increase) also significantly increased LOS. Notably, unlike the findings for men, no statistically significant effects on LOS were observed for neurological disease or injury in women (p > .05).

## Discussion

### Differences in age at admission and diagnostic patterns by sex

The study findings revealed significant differences in the age at admission and diagnostic patterns by sex among patients with alcohol-related mental and behavioral disorders. The predominant age groups were the 50s (36.6%) for male patients and the 40s (31.1%) for female patients, indicating a relatively younger age distribution among women. This trend may reflect recent sociocultural changes characterized by earlier initiation of alcohol consumption and increased drinking amounts among women [18], highlighting the need for public health interventions targeting high-risk drinking in younger women populations.

Additionally, the analysis of specific diagnoses revealed that men had a higher diagnosis rate of withdrawal state with delirium (F10.4; 17.5%, p < .001), whereas women showed nearly twice the proportion of unspecified mental disorders (F10.9; 11.5% vs. 5.7% in men, p < .001). One possible explanation is that men more frequently present with distinct acute withdrawal symptoms that clearly meet diagnostic criteria, while women may be more likely to exhibit atypical symptom presentations or receive less aggressive diagnostic approaches, leading to the higher use of unspecified codes [19]. Previous studies indicate that female patients often display non-prototypical symptom expressions that do not fit standardized criteria, including tendencies toward emotional expression, somatization of psychological distress, and limited self-reporting, which may result in ambiguous clinical assessments [20,21]. In contrast, male patients tend to manifest clear behavioral signs, such as acute withdrawal symptoms, which may facilitate the application of more specific diagnostic codes by clinicians.

Consequently, the higher use of unspecified codes (F10.9) in female patients could potentially hinder the development of appropriate treatment plans, resource linkage, and follow-up monitoring. However, caution is warranted in interpreting these findings. Since administrative hospital discharge data do not capture symptom-level clinical detail, it is not possible to definitively attribute this disparity to sex blindness or atypical symptom presentations based on the current data alone. Future studies employing structured clinical interviews and standardized diagnostic instruments would be necessary to rigorously test this hypothesis.

These diagnostic discrepancies extend beyond mere statistical differences and may serve as indicators of imbalanced clinical visibility by sex. Therefore, future improvements in diagnostic criteria and tools should incorporate sex-sensitive structured interviews and psychological assessment scales.

### Sex-specific comorbidity patterns and health inequalities

The impact of comorbidities on length of stay among patients with alcohol use disorder exhibited marked heterogeneity by sex, suggesting that alcohol-related health burdens operate differentially across sexes [6,22]. In male patients, all analyzed comorbidities demonstrated significant associations with increased length of stay, with mental disorders and trauma identified as particularly strong predictors of prolonged hospitalization. This indicates that male patients with alcohol use disorder are more likely to experience concurrent physical injuries and mental disorders, resulting in concentrated healthcare resource utilization and structural vulnerability.

In contrast, the effects of comorbidities in female patients were more selective. While mental disorders were a common factor increasing length of stay in both sexes, liver diseases, cardiovascular diseases, and diabetes emerged as primary determinants of prolonged hospitalization among women. This suggests that physical damage from alcohol use in female patients with alcohol use disorder accumulates in the form of chronic conditions, potentially leading to more sustained healthcare utilization [23].

Particularly noteworthy is that the significant effects of trauma and neurological disorders observed in men were not statistically significant in women. This suggests that, even for the same disease categories, disease severity, treatment approaches, and recovery trajectories may operate differently by sex, reflecting sex-based disparities in risk exposure and management within the healthcare system [24].

These findings clearly demonstrate that health inequalities among patients with alcohol use disorder extend beyond mere differences in prevalence, manifesting as structurally differentiated patterns of disease combinations and healthcare resource utilization by sex. Therefore, management strategies for alcohol-related mental and behavioral disorders should move beyond a one-size-fits-all approach, instead prioritizing trauma and acute neurological complication management for men, and integrated chronic disease and mental health care systems for women. This underscores that sex-tailored interventions represent a critical strategy for mitigating healthcare burdens and health disparities associated with alcohol use disorder.

### Sex disparities in determinants of length of stay

Length of stay (LOS) is a critical indicator reflecting healthcare resource consumption and the treatment burden of patients with alcohol use disorder (AUD). In this study, distinct patterns were observed in the determinants of LOS based on sex. For male patients, the presence of injury (S00–T98) was associated with a significant increase in LOS (B = 0.348, p < .0001); however, trauma did not significantly impact LOS in female patients (p = 0.942). This suggests that trauma in men may lead to more severe physical injuries or that a lack of social support systems may result in delayed recovery [6].

In contrast, mental disorders (F00–F99, excluding F10) were the strongest predictors of prolonged LOS in both sexes (B = 0.356 for men, B = 0.350 for women; p < .0001). This indicates that the comorbidity of AUD and other mental disorders increases clinical complexity and is a decisive factor in significantly extending hospitalization [25]. Notably, neurological disorders (G00–G99) significantly increased LOS in men (B = 0.267, p < .0001) but showed no significant effect in women (B = 0.107, p = .3059). This implies potential sex differences in disease severity, diagnostic tendencies, or treatment patterns. These findings demonstrate that key clinical factors affecting LOS operate differently by sex, suggesting that patient-centered inpatient management and resource allocation policies should be designed based on sex-specific characteristics.

The sex distribution of the study population was unbalanced, with 84.5% men and 15.5% women. While this may reflect the male-predominant trend of AUD, the relatively small sample size of women may have limited the statistical power in analyzing sex differences. In particular, the lack of significant effects of injuries and neurological diseases on LOS in women warrants careful interpretation. Post-hoc power analysis revealed that the female subgroup (n = 1,345) had insufficient statistical power to detect the observed effect sizes for both neurological diseases (Power = 10.6%) and injuries (Power = 28.1%), falling substantially below the conventional threshold of 80%. These findings suggest that the non-significant results in women are likely attributable to limited statistical power resulting from the smaller female sample size (15.5% of the total), rather than a true absence of effect. Future studies with larger and more balanced female samples are needed to clarify whether these associations represent genuine sex-specific differences or sampling artifacts.

Additionally, since this study relied entirely on hospital discharge codes, the LOS regression model lacks critical clinical covariates, most notably AUD severity. For instance, patients diagnosed with withdrawal state with delirium (F10.4) would be expected to have substantially longer hospital stays than those with harmful use (F10.1), independent of comorbidities. The absence of such severity indicators may have introduced residual confounding into the regression estimates. Nevertheless, this study is significant in that it reflects real-world clinical characteristics by utilizing the Korea National Hospital Discharge In-depth Injury Survey data. Future studies should consider a more balanced sex distribution and incorporate clinical severity measures to provide more precise estimates of the independent effects of comorbidities on LOS.

The study period (2014–2023) encompasses the COVID-19 pandemic (2020–2022), during which a distinct decline in discharges was observed (Fig 2). Elevated admission thresholds during this period may have introduced selection bias, as only the most severe cases were likely hospitalized, potentially skewing LOS and comorbidity estimates. The 10-year trends should therefore be interpreted with this limitation in mind. Future studies should consider a period analysis (pre- vs. during- vs. post-pandemic) to more rigorously assess the pandemic’s impact on hospitalization patterns in this population.

A further limitation relates to the generalizability of the findings. In the present study, the terms “Alcohol Use Disorder (AUD)” and “alcohol-induced mental and behavioural disorders” were used in reference to hospitalized patients only. It should be noted that hospitalized cases represent only the most severe end of the AUD spectrum, and the clinical and comorbidity profiles of this population may differ substantially from those of individuals with AUD who are managed in outpatient or community settings. Therefore, the findings of the present study should not be generalized to the broader population of individuals with AUD. Future research incorporating outpatient and community-based samples would be necessary to provide a more comprehensive understanding of the full spectrum of AUD-related health burdens.

## Conclusion

This study analyzed sex-specific characteristics, comorbidity patterns, and length of stay (LOS) determinants among patients with alcohol-related mental and behavioral disorders using data from the National Hospital Discharge In-depth Injury Survey. The results revealed that female patients were hospitalized at younger ages and exhibited higher rates of unspecified diagnoses compared to male patients, suggesting diagnostic complexity and the need for sex-sensitive healthcare approaches.

Male patients exhibited higher comorbidity rates of cardiovascular diseases and diabetes, while women showed elevated rates of mental disorders and trauma, demonstrating distinct sex differences in comorbidity patterns. Notably, comorbid mental disorders emerged as the most significant factor prolonging the length of stay (LOS) for both sexes, underscoring increased treatment complexity and the necessity for integrated mental health interventions.

Analysis of the length of stay (LOS) indicated that trauma and neurological disorders significantly extended hospitalization among men but not in women, suggesting potentially more severe alcohol-related trauma in male patients and highlighting the need for sex-tailored clinical interventions.

These findings support the development of sex-tailored treatment strategies and policies that account for differing clinical characteristics and healthcare resource utilization patterns in alcohol-related disorder management. In particular, strengthening multidisciplinary intervention systems for patients with comorbid mental disorders and implementing early intervention strategies for young and middle-aged women are urgently needed.
